# Multiple search methods for similarity-based virtual screening: analysis of search overlap and precision

**DOI:** 10.1186/1758-2946-3-29

**Published:** 2011-08-08

**Authors:** John D Holliday, Evangelos Kanoulas, Nurul Malim, Peter Willett

**Affiliations:** 1 Information School, University of Sheffield, Portobello Street, Sheffield S1 4DP, UK; 2 School of Computer Sciences, Universiti Sains Malaysia, Minden, 11800 Penang, Malaysia

## Abstract

**Background:**

Data fusion methods are widely used in virtual screening, and make the implicit assumption that the more often a molecule is retrieved in multiple similarity searches, the more likely it is to be active. This paper tests the correctness of this assumption.

**Results:**

Sets of 25 searches using either the same reference structure and 25 different similarity measures (similarity fusion) or 25 different reference structures and the same similarity measure (group fusion) show that large numbers of unique molecules are retrieved by just a single search, but that the numbers of unique molecules decrease very rapidly as more searches are considered. This rapid decrease is accompanied by a rapid increase in the fraction of those retrieved molecules that are active. There is an approximately log-log relationship between the numbers of different molecules retrieved and the number of searches carried out, and a rationale for this power-law behaviour is provided.

**Conclusions:**

Using multiple searches provides a simple way of increasing the precision of a similarity search, and thus provides a justification for the use of data fusion methods in virtual screening.

## Background

The constantly increasing costs of drug discovery have resulted in the development of many techniques for virtual screening [1-4]. One of the simplest, and most widely used, techniques is similarity searching, in which a known bioactive reference structure is searched against a database to identify the nearest-neighbour molecules, since these are the most likely to exhibit the bioactivity of interest [5-9].

A quarter of a century has passed since the first descriptions of similarity searching [10,11], but it has still not proved possible to identify some single similarity method that is consistently superior (in terms of quantitative measures of screening effectiveness such as enrichment factor or cumulative recall) to the many others that have been developed over the years [7,12,13]. Indeed, we would agree with Sheridan [6] that it is unlikely that it will ever be possible to identify such an optimal solution. There has hence been much interest in the use of *data fusion *methods, in which multiple searches are carried out and the resulting database rankings combined to yield an overall ranking (in order of decreasing probability of activity) that is the final search output presented to the user. The many studies that have been carried out have suggested that the fusion of multiple search outputs can provide an effective, and robust, alternative to conventional, single-search approaches [14]. Most of these studies have been empirical in character and have not sought to provide a theoretical rationale for the fusion procedures that have been used. There is, however, an underlying assumption that is common to all approaches to the use of data fusion for virtual screening. This assumption is that the availability of information resulting from multiple searches will increase the likelihood of detecting active molecules when compared to the use of just a single search. The assumption seems entirely reasonable but it has not, to our knowledge, been tested systematically: this article reports such a test.

The starting point for our work was a paper by Spoerri that investigated the extent to which the assumption applies when a query is matched against a database of textual documents using multiple search engines [15]. In brief, Spoerri showed that a given document was more likely to be relevant to a user's query the more search engines retrieved that document, with this likelihood increasing very rapidly as the number of search engines retrieving it increased. Spoerri called this phenomenon the Authority Effect: here, we seek to determine whether the Effect also applies in the context of similarity-based virtual screening systems, since this would provide a firm basis for the use of fusion methods.

## Results and Discussion

We have considered both of the two principal types of data fusion that have been used for virtual screening: *similarity fusion *and *group fusion *(which we refer to subsequently as SF and GF, respectively) [14]. SF involves searching a single reference structure against a database using multiple different similarity measures, and the output is obtained by combining the rankings resulting from these different measures. GF involves searching multiple reference structures against a database using a single similarity measure, and the output is obtained by combining the rankings resulting from these different reference structures. The reader should note that while we refer in this paper to the SF experiments and the GF experiments, real data fusion using either of the two approaches requires a procedure to combine the multiple ranked search outputs to give the final ranking that is presented to the searcher. Here, we have merely considered the molecules retrieved in the top-1% or top-5% of the rankings (see Experimental Methods), with no attempt being made to produce a final output ranking from the top-ranked subset of the database.

**Figure 1:**
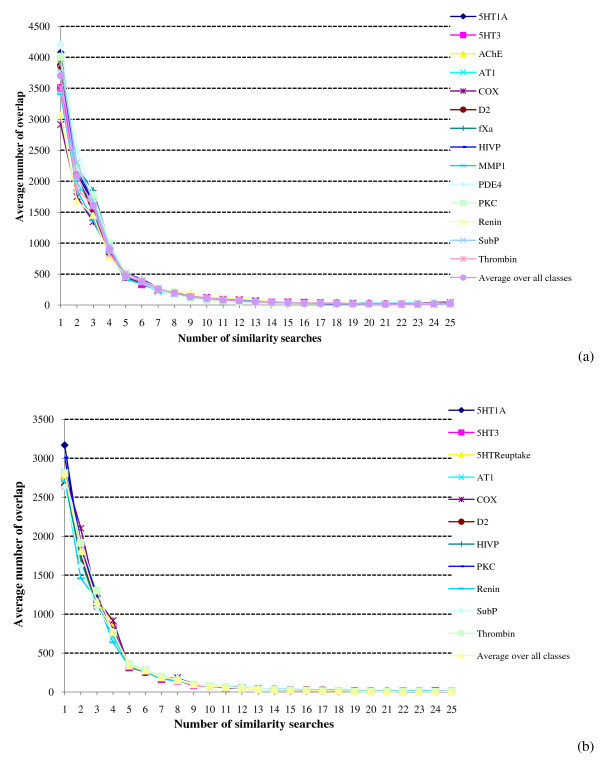
**Search overlap using similarity fusion**. Plots of the mean numbers of molecules retrieved in a given number of similarity searches for: (a) WOMBAT top-1% searches; (b) MDDR top-1% searches.

We consider first the results of the SF searches. Figure 1(a) shows the overlap plot for the WOMBAT database with a top-1% cut-off. It will be seen that the same basic pattern of behaviour is obtained for all of the activity classes, *viz *a very large number of molecules that are retrieved by just a single search, and then rapidly decreasing numbers of molecules as more searches are considered. For example, if we consider the COX-2 searches, then there were (averaged over the ten different reference structures for this activity class) 2195 different molecules retrieved once in the 25 searches, 1749 different molecules retrieved twice, 1345 different molecules retrieved thrice etc. Entirely comparable plots are obtained with the top-1% cut-off for the MDDR activity classes (Figure 1(b)) and for the top-5% searches for both datasets (data not shown). For comparison with these data, selecting WOMBAT molecules completely at random with a probability of 0.01 (for top-1% searches) in the Binomial Distribution would yield 27,128 molecules that were retrieved once; however, the numbers then drop off very rapidly so that only a single molecule would be expected to be retrieved five times and no molecules at all for greater numbers of similarity searches.

The skewed nature of the data in Figure 1 suggests that there may a power law relationship between the overlap and the number of searches, with a few observations (i.e., molecules being retrieved in the present context) occurring very frequently and the great majority occurring only once. Such relationships have been widely discussed in library and information science, where the Bradford, Lotka and Zipf distributions have been used for many years to discuss the dispersion of the scholarly literature, author productivity and word-usage frequencies respectively [16,17]. However, such relationships have been observed across the physical and social sciences: published applications include phenomena as diverse as the populations of cities, casualty figures in wars, and the sizes of lunar craters *inter alia *[18], with Benz *et al. *reviewing applications in chemoinformatics [19].

**Figure 2:**
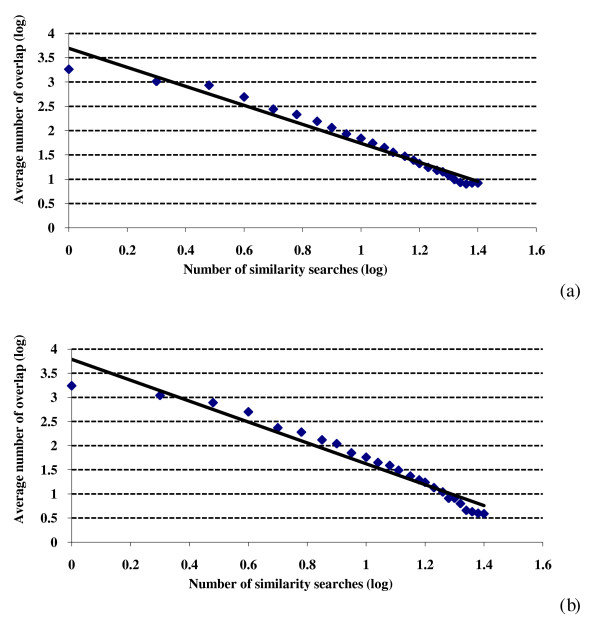
**Search overlap using similarity fusion**. Log-log plots of the mean numbers of molecules retrieved in a given number of similarity searches for: (a) WOMBAT top-1% searches; (b) MDDR top-1% searches.

A power law relationship in the current context has the general form

where *O *is the overlap (see Experimental Methods), *n *is the number of similarity searches and *a *and *b *are constants. Plotting log(*O*) against log(*n*) should then give a straight line with a slope of -*b*, and this has been tested in Figure 2 for the top-1% searches, where the overlap figures have been averaged over all of the activity classes for simplicity and ease of viewing. There are clear deviations from straight line behavior in both plots, especially at the largest and smallest numbers of searches. This is not unexpected since inspection of the log-log plots that comprise Figure four of the review by Newman [18] shows that the twelve highly disparate datasets considered there all exhibit at least some degree of curvature analogous to that observed in Figure 2. The slopes (*b*) and the *r*^2 ^values for the WOMBAT and MDDR datasets (both top-12% and top-5%) are listed in the upper part of Table 1 in the column headed 'Molecules'. It will be seen that the slopes range from -1.75 (WOMBAT top-5%) to -2.17 (MDDR top-1%) and thus cluster around the value of -2 that characterizes a classical Lotka plot [20]. Mitzenmacher has noted that log-linear plots often give results that are comparable to log-log plots in power-law studies [21]. For the SF searches in Table 1, the log-linear plots gave better *r*^2 ^values for the two top-5% results and worse values for the two top-1% values. Similarly inconsistent sets of values were obtained when the scaffold overlap and GF results were considered (*vide infra*).

**Table 1:** Slopes (*b*) and squared correlation coefficients (*r*^2^) for log-log plots of the overlap of molecules and the overlap of scaffolds using similarity fusion and group fusion

	Molecules		Scaffolds	
	
	*b*	*r*^2^	*b*	*r*^2^
	Similarity fusion			

WOMBAT top-1%	-1.98	0.966	-1.95	0.965
WOMBAT top-5%	-1.75	0.919	-1.71	0.893
MDDR top-1%	-2.17	0.959	-2.16	0.950
MDDR top-5%	-1.89	0.906	-1.89	0.881

		Group fusion		

WOMBAT top-1%	-2.49	0.957	-2.46	0.946
WOMBAT top-5%	-2.12	0.951	-2.09	0.921
MDDR top-1%	-2.32	0.952	-2.37	0.938
MDDR top-5%	-2.01	0.980	-2.00	0.968

Figures 1 and 2 consider the overlap of individual molecules. Comparable analyses were conducted in which we counted the overlap of individual ring systems, specifically the Murcko scaffolds identified by the Pipeline Pilot software. Very similar results to those above were obtained, with the numbers of distinct scaffolds again dropping off very quickly with an increase in the number of searches. The *b *and *r*^2 ^values for the scaffold log-log plots are included in the upper part of Table 1.

**Figure 3:**
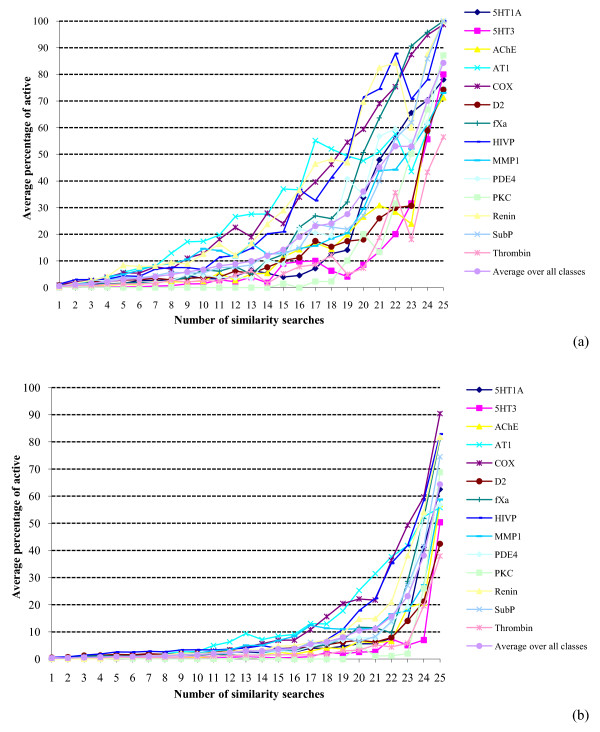
**Search precision using similarity fusion**. Plots of the percentage of the molecules retrieved in a given number of similarity searches that were active for: (a) WOMBAT top-1% searches; (b) WOMBAT top-5% searches.

**Figure 4:**
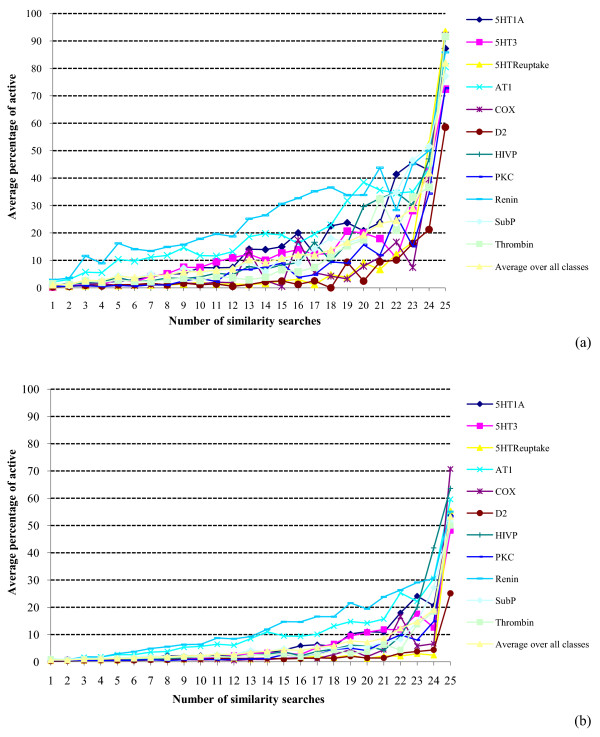
**Search precision using similarity fusion**. Plots of the percentage of the molecules retrieved in a given number of similarity searches that were active for: (a) MDDR top-1% searches; (b) MDDR top-5% searches.

**Figure 5:**
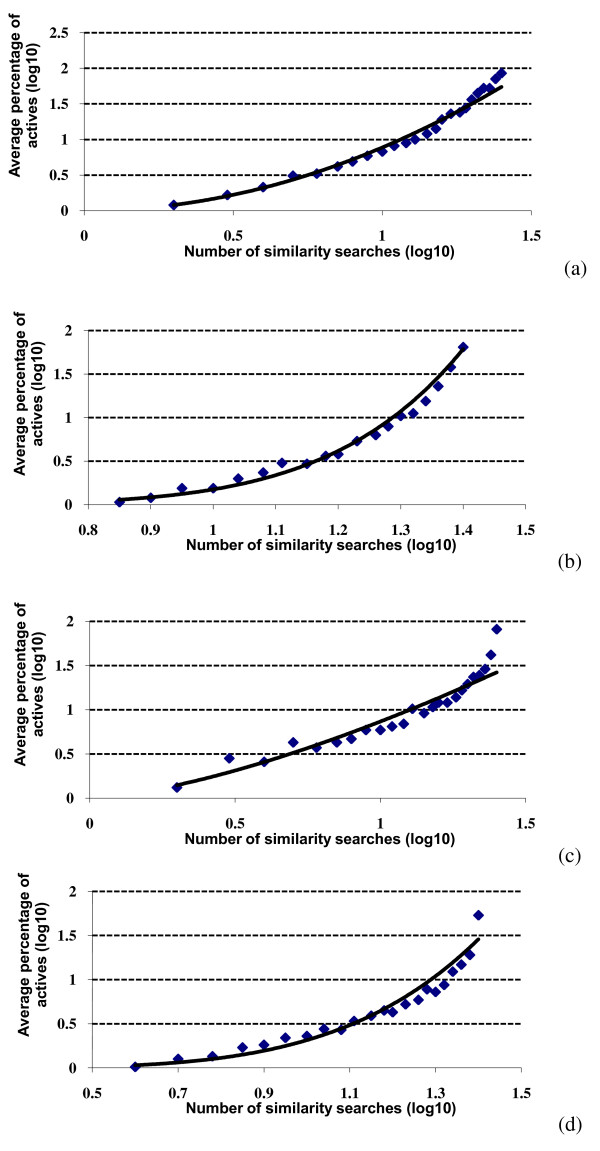
**Search precision using similarity fusion**. Log-log plots of the percentage of the molecules retrieved in a given number of similarity searches that were active for: (a) WOMBAT top-1% searches; (b) WOMBAT top-5% searches; (c) MDDR top-1% searches; (d) MDDR top-5% searches.

When applied to virtual screening, the Authority Effect would suggest that a given molecule is more likely to be active the more searches that retrieve it. From the results presented thus far, it is clear that multiple searches retrieve decreasingly small numbers of molecules; if the Effect holds then these decreasingly small numbers will contain increasingly large percentages of actives. That this enrichment occurs in practice is clearly demonstrated in Figures 3 and 4. There are often marked differences between the various activity classes comprising a dataset but the plots are at one in showing that the precision (see Experimental section) is very low for molecules retrieved by just a few searches but that it then increases very rapidly as the number of searches moves towards the maximum. As in the overlap experiments, the skewed nature of the data suggests that a power law relationship may be appropriate to describe the relationship. Averaging over all of the activity classes, the precision figures are shown as log-log plots in Figure 5. The plots all curve upwards to the right: fitting the log-log data to power and exponential trends, the former always gave the better fit, with the continuous curves in the figures representing a cubic relationship. Comparable results to those shown in Figures 3, 4, 5 were again obtained when we considered the active molecules' scaffolds that were retrieved, rather than the active molecules that were retrieved.

We hence conclude that a molecule is more likely to be active the more frequently it is retrieved when multiple similarity measures are available for carrying out a similarity search for a bioactive reference structure. The Authority Effect would thus appear to hold, at least for the datasets and similarity measures used here.

**Figure 6:**
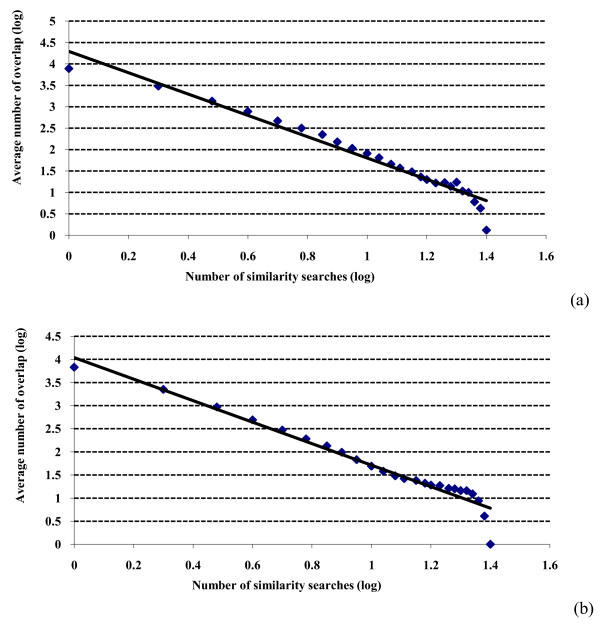
**Search overlap using group fusion**. Log-log plots of the mean numbers of molecules retrieved in a given number of similarity searches for: (a) WOMBAT top-1% searches; (b) MDDR top-1% searches.

Turning now to the GF searches, the overlap plots that were obtained are very similar in form to those shown in Figures 1 and 2, and we have hence included just the top-1% log-log plots in Figure 6. The *b *and *r*^2 ^values for these plots are included in the lower part of Table 1, and it will be seen that the magnitudes of the slopes are larger than in the upper part of this table, i.e., the numbers of molecules retrieved drops off more rapidly than in the similarity fusion searches. However, this drop-off is from a much larger starting point, as can be seen by comparing the intercepts on the *y*-axis in, e.g., Figures 2(a) and 6(a), i.e., the single similarity measure and 25 reference structures in the GF search identify a notably larger number of molecules than the 25 similarity measures and single reference structure in the SF search. This behaviour is detailed in Table 2, which shows the mean numbers of common molecules and common scaffolds for SF and GF searches using 1, 5, 10, 15, 20 and 25 similarity searches.

**Table 2:** Numbers of common molecules and of common scaffolds in similarity fusion (SF) and group fusion (GF) WOMBAT top-1% searches

Similarity searches	Common molecules		Common scaffolds	
	
	SF	GF	SF	GF
1	3705	7759	1821	3771
5	473	469	277	290
10	107	81	69	51
15	37	23	25	15
20	20	18	12	9
25	24	1	8	1

We believe that there are two factors that may explain the observed difference between SF and GF. First, the very different natures of the two types of search. In an SF search, the same reference structure is used in all 25 searches. Now, the substructures present within that structure are encoded in different ways by the five different fingerprints, and those encodings are processed in different ways by the five different similarity coefficients; however, it is the same basic structural information that is being used in each and every search. In the GF searches, conversely, a totally different reference structure (and hence different structural information) is used in each search. Second, some of the similarity measure components are quite closely related to each other; thus the Tanimoto and cosine coefficients are known to give very similar (though not monotonic) rankings [22], and the Unity and Daylight fingerprints use a similar fragment encoding scheme. Thus, not only is the same basic structural information being used for all the SF searches, but in some cases this information is being processed in a similar manner. Taking these two effects together, the top-ranked molecules resulting from the SF searches hence have a greater degree of commonality than the top-ranked molecules from the GF searches, making it relatively easier for a molecule to be retrieved multiple times using SF (and relatively more difficult using GF). In like vein, a still more steeply angled plot (albeit one that is not based on a log-log relationship) is obtained when searching is simulated by drawing molecules at random using the Binomial Distribution, resulting in sets of molecules having minimal structural commonality.

**Figure 7:**
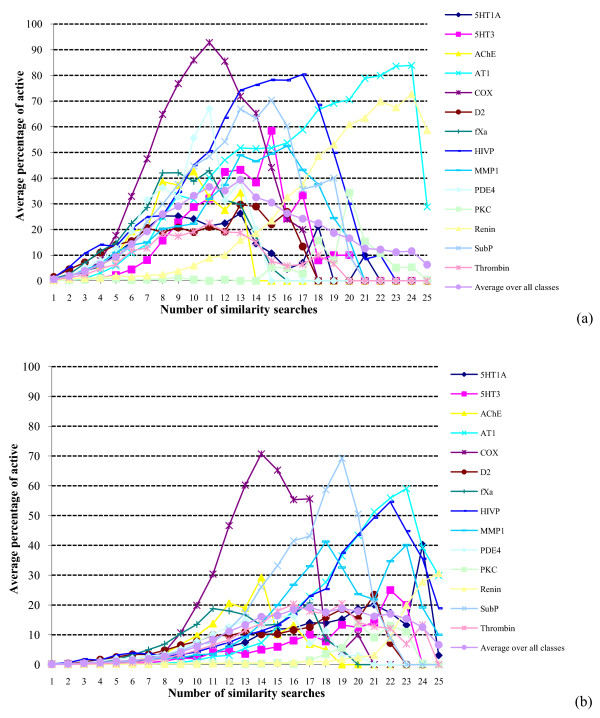
**Search precision using group fusion**. Plots of the percentage of the molecules retrieved in a given number of similarity searches that were active for: (a) WOMBAT top-1% searches; (b) WOMBAT top-5% searches.

**Figure 8:**
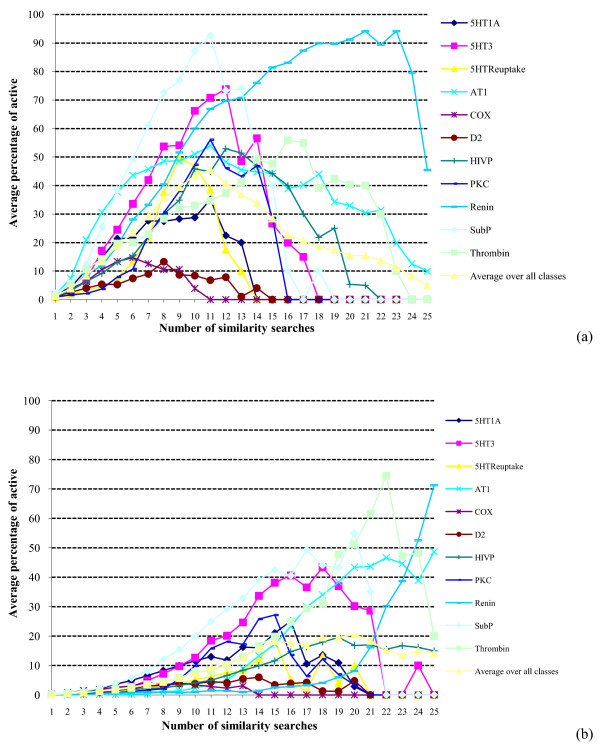
**Search precision using group fusion**. Plots of the percentage of the molecules retrieved in a given number of similarity searches that were active for: (a) MDDR top-1% searches; (b) MDDR top-5% searches.

**Figure 9:**
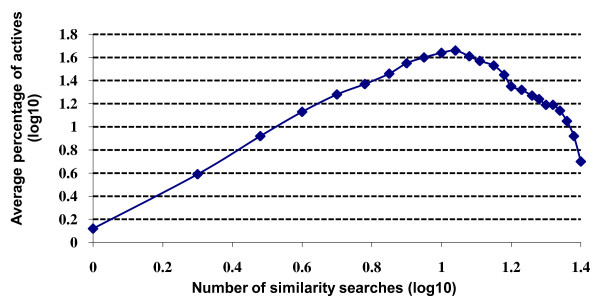
**Search precision using group fusion**. Log-log plot of the percentage of the molecules retrieved in a given number of similarity searches that were active for MDDR top-1% searches.

The differences between the two types of fusion are still more marked when we consider the precision of the GF searches, as can be seen by comparing the results in Figures 7 and 8 with those in Figures 3 and 4. The general GF trend is for the precision to rise steeply (as in the SF searches) but then to fall rapidly away, giving an inverted bell-shape rather than the constantly increasing plots observed previously (see also the log-log plot for the MDDR top-1% data in Figure 9). The low precision values observed towards the right-hand parts of the Figures 7 and 8 plots follow naturally from the discussion above since if the 25 reference structures in a GF search are quite disparate then it is unlikely that many, or even any, molecules will be retrieved by large numbers of these reference structures. The precision (when averaged over the ten sets of GF searches for each activity class) is hence expected to be low, and there is some evidence to support this view from consideration of the individual activity classes. Specifically, there is a tendency for the more homogeneous activity classes (such as the renin inhibitors) to exhibit their maximum precision at larger numbers of searches than for the less homogeneous (i.e., more heterogeneous) activity classes, where we approximate the homogeneity of an activity class by the mean pair-wise similarity when averaged across all the pairs of molecules in that class. For example, consider the MDDR top-1% GF searches. We have ranked the eleven activity classes in order of decreasing mean pair-wise similarity and noted for each such class the number of similarity searches (in brackets) that gives the maximum precision. The resulting order is: Renin (23) > HIVP (12) > Thrombin (16) > AT1 (11) > SubP (11) > 5HT3 (12) > 5HTReuptake (9) > D2 (8) > 5HT1A (11) > PKC (11) > COX (6). Thus the differences in behavior between the GF and SF searches tend to increase the more diverse the activity class that is being sought, i.e., the more disparate the reference structures that are used for the searches. Support for this view comes from previous studies by Hert *et al. *[23] and by Whittle *et al. *[24], who showed that GF, using the MAX and SUM fusion rules respectively, gave comparable levels of screening effectiveness to conventional similarity searching (and hence, by implication, to similarity fusion) when structurally homogeneous activity classes were searched; however, there were noticeable differences in screening effectiveness (with GF the superior approach) when more heterogeneous classes were searched.

We hence conclude that the Authority Effect applies to GF searches when the reference structures are structurally quite similar; when this is not the case, it is applicable when relatively small numbers of reference structures are used, i.e., when meaningful numbers of molecules are being retrieved in all of the searches. It should be emphasized that this does not mean that GF is in some way inferior to SF as a technique for ligand-based virtual screening. First, the discussion here has focussed on the numbers of active molecules that are retrieved, without consideration of their diversity, and the previous studies mentioned above demonstrated the applicability of GF when structurally diverse molecules are sought [23,24]. Second, it must be remembered that whilst we refer to SF and GF, practical implementations of these techniques entail a subsequent step in which a fusion rule combines the sets of nearest neighbours from the individual searches. Finally, if 25 different, active reference structures were available, one should probably be using a more sophisticated, machine learning method [25] for database screening, e.g., a naive Bayesian classifier or a support vector machine, rather than simple, similarity-based approaches.

The results above show that Spoerri's Authority Effect holds - to some extent - for the chemical datasets and biological activity classes considered here. Specifically, a molecule is more likely to be active the more frequently it is retrieved in multiple similarity searches using a single reference structure or in multiple similarity searches using structurally similar multiple reference structures. This observation hence provides a justification for the use of data fusion methods in ligand-based virtual screening. In saying that, we must emphasise that our experiments have been conducted specifically to investigate the Authority Effect, and that rather different procedures are normally applied when data fusion procedures are used in operational virtual screening systems. For example, a common approach to GF is to use the MAX (or 1-NN) fusion rule, where the similarity for a database structure is taken to be the maximum of the similarities between that structure and each of the reference structures. Whittle *et al. *have shown that the numbers of retrieved actives increase approximately monotonically with the number of GF reference structures even when many of them are employed (see Figure six in Ref. [24]). Again, if one were to use SF in practice, one would choose similarity measures that differed in character, as exemplified by the work of Muchmore *et al. *on belief theory [26], rather than the similar 2D fingerprint measures used here. Thus, while the results that we have presented provide a basis for the use of data fusion methods in principle, they do not provide a guide as to the effectiveness of any specific fusion method in practice.

It would clearly be desirable if we could not only demonstrate, but also rationalize, the frequency plots that we have presented. There has recently been interest in the underlying mathematical models that could generate power law distributions (see, e.g., [18,21]). Mitzenmacher has identified five broad types of generative model, and applied them to the analysis of both log-log and log-normal distributions [21]. In what follows, we apply a modification of one of his types - which he refers to as 'preferential attachment' - to the analysis of our virtual screening data.

Assume that there are *n *similarity search methods available, each of which models the possible activity of a molecule in a similar manner. Without loss of generality, assume also that the search methods for a given query (i.e., a single reference structure in similarity fusion or a set of reference structures in group fusion) are run sequentially. At each time step, a search is conducted of the *M *molecules in a database and a set of *m *possibly active molecules is returned (e.g., those in the top-5% of the ranking resulting from that search method). Thus, at time step 1, the first search is run and a set of *m *potentially active molecules is returned; at time step 2, a second search is run and another set of *m *possibly active molecules is returned, and so on. We now make the following assumption: that the second search returns the molecules that have been already returned by the first search with some probability proportional to γ (γ <1) while the rest of the molecules are returned with a probability proportional to (1- γ). Then, when the third search is conducted, a molecule is retrieved with probability proportional to the number of searches that have already returned that molecule. We are using here retrieval methods that are basically very similar (e.g., all using the same basic 2D substructural components and closely related association coefficients in a similarity fusion search), and it is hence not unreasonable to assume that a molecule satisfying the search criterion for one method is also likely to satisfy the criteria for other, related methods. If the different search methods are all equally similar to each other then a single γ is able to capture this similarity independently of the order of the methods used for searching the database. This is the strongest assumption we make here.

At the end of all the *n *searches, a total of n*m molecules will have been retrieved (though some of these will have been retrieved more than once). Let *O_s _*denote the fraction of molecules returned by exactly *s *searches (i.e. the overlap between *s *similarity searches): we now demonstrate that *O_s _*follows a power-law distribution. However, before providing a mathematical derivation of the distribution, we shall illustrate the approach using the example of four searches each retrieving three molecules as shown in Table 3. The set of molecules returned by three searches is {C}, while the set of molecules returned by two searches is {A, B, D}. If the current search (Search 5) returns any of A, B or D then the size of the set of molecules returned by three searches will increase by one. The chances of one of the three molecules being selected by Search 5 is γ*(2*3/12), since out of the 12 molecules already returned there are 2*3 = 6 instances of molecules already returned twice. If the current search returns C then the size of the set of molecules returned by exactly three searches will decrease by one since C now belongs to the set of molecules returned by exactly four searches. The chance that the molecule C is returned is γ*(3*1/12) since out of the 12 molecules already returned there are 3*1 = 3 instances of molecules already returned thrice. If the growth of the set of molecules returned *s *times can be expressed mathematically then we shall be able to model the distribution of the fraction *O_s_*, as we now demonstrate. In saying that, the reader should note that the following derivation excludes the special case of *s *= 1: this is not only to simplify the explanation but also because *s *= 1 is the extreme end of the distribution, corresponding to molecules retrieved just once in any of the *n *searches and thus unlikely to be of practical interest in a screening context. The full derivation is presented by Mitzenmacher [21].

**Table 3:** Sets of three molecules retrieved in each of four searches

Search 1	Search 2	Search 3	Search 4	Search 5
{A, B, C}	{B, C, D}	{C, D, E}	{F, G, H}	?

Let *X_s_*(*t*) be a random variable describing the number of molecules returned by *s *searches at time step *t*. Then for *s *≥ 2 the increase in *X_s_*(*t*) is described by the following formula

This is the probability that the current search returns one of the molecules retrieved in *s*-1 of the previous searches. The denominator m*t is the total number of all retrieved molecules up to time step *t*, (*s*-1)*X_*s*-1 _is the total number of instances already retrieved *s*-1 times, and thus (*s*-1)*X_*s*-1_/m*t is the fraction of the complete set of retrieved molecules that has been previously retrieved by *s *searches. Since the current search returns *m *molecules, the probability that a molecule is retrieved given that it has already been retrieved *s*-1 times is hence

and

The decrease of *X_s_*(*t*) is described by the following formula

hence it is equal to the probability that the current search returns one of the molecules previously retrieved by *s *searches. Here, s*X_s _is the total number of molecules already retrieved by *s *searches, and thus s*X_s_/m*t is the fraction of the complete set of retrieved molecules that has been previously retrieved by *s *searches. The probability that a molecule is retrieved given that it has already been retrieved *s *times is hence

The growth of *X_s _*is hence given approximately by

After all *n *searches have been executed *X_s_*(*t*) = O_s_*m*t, i.e., the molecules retrieved by *s *searches constitute a fraction *O_s _*of all the molecules retrieved. In the general case (for *s *≥ 2)

Solving for the fraction of molecules returned by *s *searches gives

For large *s*, γ*s+1 ~ γ*s and thus,

Asymptotically, for the above to hold, we have  for some constant *a*, giving a power law distribution for the fraction *O_s _*and hence a rationale for the behavior observed in the MDDR and WOMBAT searches (see Figures 1, 2 and 6).

The reader should note that *b*= -(1+1/*γ*) can only give rise to exponents (slopes) that are less than -2, i.e. *b *= -(1+1/*γ*) ≤ -2, for 0 <*γ *≤ 1, so that some of the exponents (slopes) shown in the upper part of Table 1 cannot be explained by the proposed model. These are the slopes empirically derived from the data for the molecules and scaffolds using similarity fusion, regarding which we make two comments. First, the goodness of fit, as measured by *r*^2^, is not as high as the goodness of fit for the rest of the empirical data, suggesting that the slope *b *may not be accurate enough. Second, the number of searches may not be large enough for accurate use of the approximation γ*s+1 ~ γ*s in the derivation. In particular, using the formula before this approximation and simulating the overlaps for different values of *γ *based on the formulae above we obtain: for *γ *= 0.9, *b *= -1.923>-2, while for *γ *= 0.99, *b *= -1.845>-2. This can explain most of the slopes in Table 1 with the exception of those for WOMBAT top-5%.

It must be emphasized that this derivation considers only the overlap of the search outputs and says nothing about the precision of the searches. There is, however, an analogy that suggests one way in which the precision distributions might be modeled in future work. The overlap plots show that there is a distinct lack of consistency, i.e., that the different search methods generally retrieve very different sets of molecules. This situation has also been shown to pertain in many analogous retrieval contexts, such as the assignment of indexing terms [27], the creation of links in hypertext systems [28] and the selection of search strategies [29]*inter alia*. In particular, it has been suggested that while indexers often differ considerably as to which indexing terms should be assigned to documents, where there is a high degree of consistency then this should result in enhanced search effectiveness. Whilst generally dubious of the correctness of this suggestion in practice, Cooper has shown, using a highly simplified model of the retrieval process, that effectiveness gains are obtainable in principle [30], and it may be that analogous procedures could be applied to the modeling of the search results in Figures 3, 4, 7 and 8.

## Conclusions

Data fusion, or consensus, methods are being increasingly used to combine the rankings that result from multiple virtual screening searches, with the hope that the combined ranking will contain a greater number of active molecules than will the original rankings. Our experiments with the MDDR and WOMBAT datasets demonstrate that different ranking methods result in markedly different sets of retrieved molecules, with the numbers of retrieved molecules common across a set of search outputs dropping off rapidly as the number of searches is increased. Specifically, we find an inverse log-log relationship between the numbers of searches carried out and the numbers of molecules common to those searches, with this power-law relationship being obtained when both similarity fusion and group fusion consensus approaches are used. However, whilst the numbers of retrieved molecules in common drop away very rapidly as more searches are carried out, the fraction of those that are active increases in the case of similarity fusion, or increases to a maximum before falling away in the case of group fusion. We also describe a generative model for the overlap between different screening searches, which provides a quantitative basis for the observed power-law behaviour. Thus, while the work presented here does not immediately suggest any new way of carrying out virtual screening, it does provide a rationale, both empirical and theoretical, for the use of a practice that is widely used in virtual screening, i.e. data fusion.

## Experimental Methods

Testing the applicability of Spoerri's Authority Effect to virtual screening requires test datasets containing molecules of known (in)activity in one or more bioassays, and a range of different measures that can be used to carry out similarity searches on those datasets. Two separate datasets were used, these being the MDL Drug Data Report (MDDR) and World of Molecular Bioactivity (WOMBAT) databases. The versions used here were those that we have employed in many previous studies of virtual screening in this laboratory and that are described in detail by Arif *et al. *[31]. In brief, the MDDR file contained 102,535 molecules, with searches being carried out for 11 activity classes; and the WOMBAT file contained 138,127 molecules, with searches being carried out for 14 activity classes. The databases were searched using the two types of data fusion: similarity fusion (SF) and group fusion (GF).

In the SF experiments, ten compounds were randomly selected from each of the chosen activity classes to act as the reference structure for a similarity search, with each reference structure being searched using a total of 25 different similarity measures. These were obtained by combining five different 2D binary fingerprints with five different similarity coefficients. The 2D fingerprints used for describing the reference structure and the database structures were 166-bit MDL Keys, 1052-bit BCI bit-strings, 2048-bit Daylight fingerprints, 998-bit Unity fingerprints, and 1024-bit Pipeline Pilot ECFP_4 fingerprints. The similarity coefficients used to measure the similarity between the reference structure's fingerprint and the database structures' fingerprints were the Cosine, Forbes, Russell-Rao, Simple Match and Tanimoto coefficients [32]. In the GF experiments, 25 compounds were randomly selected from each of the chosen activity classes to act as the reference structures, and these were then searched using ECFP_4 fingerprints and the Tanimoto coefficient; ten such sets of 25 compounds were used for each activity class.

Given a specific reference structure, a similarity search was carried out using each of the different similarity measures in turn, yielding a total of 25 rankings (SF) or a similarity search was carried out using the ECFP_4/Tanimoto measure for each of the 25 reference structures (GF). A threshold was then applied to each of the resulting database rankings to obtain the nearest neighbours of the reference structure, i.e., the top-ranked database structures. The thresholds here were the top-1% and the top-5% of the rankings. For each of the molecules in a database, a note was made as to the number of times that it was identified as a nearest neighbour, so that each database structure had an associated integer value between 0 (meaning that it was retrieved in none of the searches) and 25 (meaning that it was retrieved in all of the searches). The resulting sets of integers, which are independent of the order in which the searches were carried out, were then processed to identify the *search overlap *and the *search precision*: the overlap measures the extent of the overlap between the search outputs, in terms of the numbers of molecules retrieved by some specific number of different searches; and the precision measures the percentage of the molecules retrieved by some specific number of different searches that are active. The nearest-neighbour data was collected for each reference structure in turn, and the results for each activity class were obtained by averaging over the set of ten searches for that class (and some of the results that are discussed are averaged over the set of 11 (for MDDR) or 14 (for WOMBAT) activity classes).

## Competing interests

The authors declare that they have no competing interests.

## Authors' contributions

NM carried out all of the virtual screening experiments with assistance from JH. EK carried out the mathematical power-law analysis. PW conceived the study, participated in its design and coordination, and drafted the manuscript with assistance from JH, NK and NM. All authors read and approved the final manuscript.
